# Recurrent anti-NMDAR encephalitis during pregnancy combined with two antibodies positive

**DOI:** 10.1007/s00737-021-01124-5

**Published:** 2021-04-09

**Authors:** Hong Liu, Xiu Chen

**Affiliations:** Department of Neurology, The Affiliated Hospital of Southwest Medical University, Luzhou, 646000 China

**Keywords:** Anti-N-methyl-d-aspartate receptor (NMDAR) encephalitis, Double-antibody positive, Recurrent, Pregnancy

## Abstract

Anti-N-methyl-d-aspartate receptor (NMDAR) encephalitis is an autoimmune synaptic encephalitis likely mediated by neuronal surface antibody. Clinically, it is characterized by a variety of neurological and psychiatric symptoms, predominantly affecting young women. Recurrent anti-NMDAR cases combined with double-antibody positive during pregnancy have not been reported. We report a 19-year-old pregnant woman with recurrent anti-NMDAR encephalitis and double-antibody positive. Through our case report and a review of the literature, we hope to heighten an awareness of anti-NMDAR encephalitis, particularly in a pregnant setting.

## Introduction

Anti-NMDAR encephalitis is an autoimmune synaptic encephalittis likely mediated by neuronal surface antibody against the NR1 subunit of the receptor, which was firstly described in four young women with ovarian teratoma in 2005 (Dalmau et al. 2011). At present, although the underlying pathogenesis about the disorder has not been fully explained, more and more data show that antibodies developed in response to a number of possible stimuli (e.g. tumour, infection), likely form cross-reaction with synaptic proteins, most commonly the NMDAR. Patients usually present with acute or subacute neuropsychiatric symptoms, epilepsy, cognitive impairment, disturbance of consciousness, and autonomic nervous instability. When severe, the disorder would be life-threatening and intensive care treatment is desperately needed. To the best of our knowledge, anti-NMDAR encephalitis occurring during pregnancy is infrequent. Until now, based on the data in PubMed from 2007 to 2020, just only 28 cases have been published in public. No double-antibody positive recurrent anti-NMDAR encephalitis in pregnancy has been reported. Through our case report and a review of the literature, we hope to heighten an awareness of anti-NMDAR encephalitis, particularly in a pregnant setting.

## Case

A 19-year-old pregnant woman developed acute psychiatric symptoms and oral-face-brachial dystonia at the 8th weeks of her third pregnancy. Physical examination of the nervous system revealed blurred consciousness, restlessness, slow reaction, poor memory and orientation, involuntary movement of the oral and facial arms, and high muscle tension of the limbs. Other physical examinations showed no positive signs. Barthel scale score was 40 points. What’s interesting is that when she was at the 10th weeks of her first pregnancy, this patient encountered severely neuropsychiatric symptoms similar to this time, but no seizures. Her blood culture during hospitalization turned out to be *Escherichia coli*. Despite continuous treatment, her clinical response did not improve significantly until the pregnancy was terminated at the 15th weeks of gestation. The patient fully recovered after taking olanzapine for 3 months. During the second pregnancy, she was absolutely normal and had given birth to a healthy boy.

**Fig. 1:**
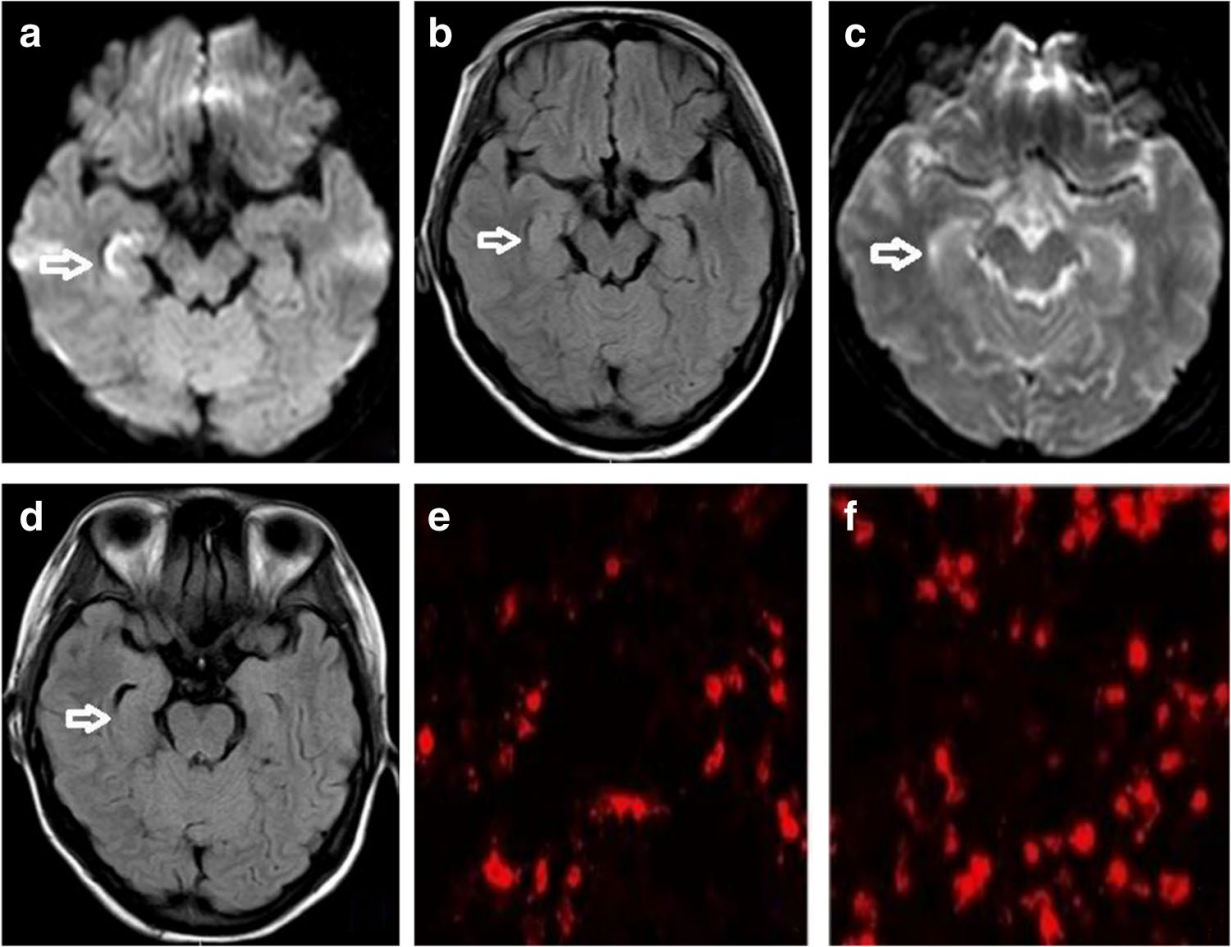
Symptoms and auxiliary examination in a patient with anti-NMDAR encephalitis. **a** Diffusion-weighted imaging of right hippocampal hyperintensity on the first MRI. **b** High signal intensity in right hippocampus of patients with FLAIR sequence on the first MRI. **c** The sequence of DWI in the second reexamination of MRI. **d** The sequence of FLAIR in the second reexamination of MRI. **e** The titer of anti-NMDAR antibody is 1:32. **f** The titer of anti-NMDAR antibody is 1:320

Lumbar puncture examination was completed upon admission. Cerebrospinal fluid pressure was 150 cm H2O. CSF analysis showed white cells 47 × 10^6^/L (normal value < 8 × 10^6^/L), mononuclear cells account for 95%. The levels of protein, glucose and chloride were normal. No abnormalities were found in bacterial culture, fungi, acid-fast bacilli and cryptococcus. Gynecological Doppler showed the following: intrauterine single live fetus, breast, pelvic cavity, ovary, fallopian tube no abnormality. Admission routine tests, thyroid function, tumor biomarkers, and autoantibody profiles were normal. Brain magnetic resonance imaging illustrated Flair/DWI sequences hyperintense signal in the right hippocampus (Fig. 1). EEG patterns revealed bilateral diffuse and persistent theta-delta slow-wave. The cerebrospinal fluid and serum were sent to Beijing Hearst Medical Laboratory for autoimmune encephalitis-related antibodies. The detection results showed anti-NMDAR-IgG 1:32 in CSF (Fig. 1) and 1:1000 in serum. The paraneoplasm-related antibody amphiphysin in serum was positive, while that one in CSF was negative, thus confirming the diagnosis of anti-NMDAR encephalitis. First-line immunotherapy is recommended according to the management guidelines for autoimmune encephalitis. The patient received a course of intravenous immunoglobulin (0.4 mg/kg/day) and intravenous pulse methylprednisolone (1000 mg/day) for 5 days, and methylprednisolone is gradually reduced to oral dose. Considering the possible harmful effects of some anti-epileptic agent on the fetus, we choose the lamotrigine to control episodic dystonia. After received immunotherapy, the patient’s mental state was slightly improved, but there were still have oral-face-brachial dystonia and obvious cognitive impairment, and the patient was unable to take care of herself in daily life. She often refused to eat and did not sleep at night. However, the patient revisited the head MRI again; we found that the abnormal signal disappeared in the right hippocampus (Fig. 1). Barthel scale score was 70 points. After her husband weighed the pros and cons, she induced abortion to terminate the pregnancy at 15th weeks. The presence of a tumor in her body has been the focus of our attention since the antibody amphiphysin in serum was positive. According to the general examination results, no tumor was found, especially teratoma. Prednisone acetate, lamotrigine and olanzapine were continued orally after discharge. However, the patient refused to receive second-line immunosuppressive therapy due to economic reasons. Follow-up for 3 months, the patient’s mental state was basically normal, without epileptic seizure, the basic diary life activities are basically self-care, but there are still obvious dyscalculia and memory disorders. Barthel scale score was 85 points. MMSE scale score is 14 points (her education background is primary school education). Follow-up for 1 year, the patient was recovered without any sequelae. The MMSE scale score is 20 points.

Two years later, at the 10th weeks of her 4th pregnancy, she developed similar psychiatric symptoms and epileptic symptoms. Barthel scale score was 40 points. After admission, she completed related laboratory tests. Brain MRI was normal. Transvaginal ultrasonography showed no teratoma or mass. EEG showed overall slow wave activity. CSF analysis showed white cells 56 × 10^6^/L, mononuclear cells account for 96%. The level of protein, glucose and chloride were normal. Herpes simplex and varicella-zoster virus PCR and fungal and acid-fast bacillus smear and cultures were negative. Since anti-NMDAR encephalitis had been diagnosed previously in this patient with similar symptoms, we suspected the relapse of anti-NMDAR encephalitis. CSF and serum samples were sent for anti-NMDAR antibody analysis. It was detected that the anti-NMDAR-IgG was 1:32 in CSF and 1:320 in serum (Fig. 1). At the same time, the anti-AMPAP-IgG was found to be 1:3.2 in CSF and 1:10 in serum. No abnormalities were found in systemic tumor screening. The patient was treated with a first-line regimen of immunoglobulin (0.4 mg/kg/day) and intravenous pulse methylprednisolone (1000 mg/day) for 5 days. Levetiracetam was chosen to control epilepsy because it had less effect on the fetus. Despite continuous treatment, her clinical response did not improve significantly until the pregnancy was terminated at the 15th weeks of gestation. Unfortunately, she still could not afford second-line immunotherapy. At the time of discharge, the patient’s mental behavior was slightly improved, epilepsy occasionally occurred, and cognitive function was significantly impaired, especially memory and orientation. She continued to take hormones, levetiracetam, and olanzapine outside the hospital. Follow-up for 3 months, she basically recovered and only had poor memory and mild cognitive impairment. Barthel scale score was 80 points. The MMSE scale score was 14 points. Written informed consent of the patient is required for the publication of case reports and associated images.

## Discussion

Autoimmune encephalitis (AE), accounting for 10–20% of encephalitis, is a kind of immune-mediated encephalitis, which may be associated with the presence of specific autoantibodies. Anti-NMDAR encephalitis is the most common subtype, accounting for about 80% of AE (Steiner et al. 2020). NMDAR is an indispensable part of the nervous system. A growing body of studies have identified that neurological system diseases, such as cerebrovascular diseases, Alzheimer’s disease, amyotrophic lateral sclerosis, Parkinson’s disease, and multiple sclerosis, have altered the activation or expression of NMDAR, which would aggravate nerve injury by increasing calcium influx and regulating downstream signaling molecules (Parsons and Raymond 2014). In addition, NMDAR might have an effect on brain development, and glutamate dysfunction would play an important role in the occurrence of schizophrenia (Funayama et al. 2018). Excessive excitation of NMDA receptors could cause excitotoxicity of nerve cells and lead to anti-NMDAR encephalitis (Dalmau et al. 2008).

Clinical features of anti-NMDAR encephalitis consisted of psychiatric symptoms, neurological symptoms (epilepsy, motor disorder, memory disorder and consciousness disorder) and autonomic instability (Dalmau et al. 2008). MRI usually shows normal or only slight changes. Hyperintensity in T2/Flair phases in the hippocampus or temporal lobes (unilateral or bilateral) is typical for MRI. These lesions are usually mild or transient with subtle contrast. In our case, the first cranial MRI showed a Flair/DWI hyperintensity signal in the right hippocampus, which disappeared after 3 weeks. Electroencephalography is usually characterized by nonspecific, general slow waves, epileptic waves, states, and periodic lateralized epileptoid discharges. Delta brush (a broad rhythmic Delta activity with rapid movement superposition) is characteristic of NMDAR encephalitis (Florance et al. 2009). Confirmation of diagnosis will depend on anti-NMDAR antibody IgG in cerebrospinal fluid and serum.

The treatment of AE includes immunotherapy, management of seizure and psychiatry, supportive and rehabilitation. In tumor-associated AE, surgical treatment should be initiated as soon as possible. Notably, in the case of anti-NMDAR encephalitis co-existing teratoma, early removal of the teratoma served as a first-line therapy, with rapid institution of immunosuppressive treatment following the surgery. First-line immunotherapy involves methylprednisolone, IVIg and plasma exchange alone or in combination. Recent data maintained that over half of patients respond to first-line immunotherapy within 4 weeks. Second-line immunotherapy is comprised of rituximab and cyclophosphamide. When the patient’s response to the first-line therapy was deemed to be partial, second-line immunosuppressive treatment should be implemented in this scenario. Maintenance therapy during clinical improvement includes mycophenolate and azathioprine. Alternative therapy contains tocilizumab and low-dose interleukin-2. Joubert B’s study reported that 50–70% of patients received steroids and immunoglobulin therapy during pregnancy and one-third of patients received plasma exchange, individual patients received rituximab or cyclophosphamide second-line immunotherapy (Joubert et al. 2020).

Dalmau (Dalmau et al. 2011) described the recurrence of anti-NMDAR encephalitis as a new psychiatric or neurological syndrome that could not be explained by other causes after the improvement of immunotherapy symptoms or a few spontaneous remissions. Recurrent anti-NMDAR encephalitis is associated with incomplete treatment of anti-NMDAR encephalitis, including patients who did not receive immunotherapy (Simabukuro et al. 2015) for the first time, who did not receive adequate immunotherapy (Titulaer et al. 2013), and who did not find or resect tumors (Schein et al. 2017). The recurrence rate of anti-NMDAR encephalitis was relatively low (about 15–25%), while the recurrence rate of multiple encephalitis was 33% (Dalmau et al. 2011). In our case, anti-NMDAR IgG was positive in the 3rd and 4th pregnancies, which was considered to be anti-NMDAR encephalitis recurrence. No tumor was found in two general screening tests, and the patient’s symptoms improved after the termination of pregnancy, which is speculated that pregnancy may be the main reason for its recurrence. The association between pregnancy and anti-NMDAR encephalitis is unclear. Anti-NMDAR encephalitis is an autoimmune disease caused by organ and tissue damage mediated by antibodies or T cells. Previous studies have shown that the pathogenesis may be related to the immune regulation of sex hormones (Bouman et al. 2005), the gene coding of X and Y chromosomes (Bellott et al. 2014), pregnancy-related specific immune basis (Østensen et al. 2012), and genetic and environmental factors(Borchers et al. 2010). The levels of estrogen and progesterone change at different stages of pregnancy. Estrogen can stimulate the survival of B cells and the production of antibodies, inhibit the expansion of T cells(Peery et al. 2012), and exacerbate the autoimmune disorder. The complex immune response of the mother to the potential antigen of “half-self, half-non-self” in the fetus (Mor and Cardenas 2010) increases the incidence of autoimmune diseases. Pregnancy is prone to depression, irritability and other emotional disorders (Fornaro et al. 2020), and there are potential mental factors. Therefore, pregnancy may be an inducer of anti-NMDAR encephalitis and other autoimmune diseases.

Interestingly, in addition to positive anti-NMDAR-IgG, our case was also associated with other positive antibodies. The first was positive for anti-NMDAR-IgG and amphiphysin. However, the second time was positive for anti-NMDAR IgG and anti-AMPAP IgG. Autoimmune antibodies can be separated into two kinds of types: antibodies against intracellular proteins (e.g. anti-Hu, Yo, Ri, amphiphysin), and antibodies acting with neuronal cell-surface receptors or synaptic proteins (e.g. anti-NMDAR-IgG, GABABR-IgG, AMPAP-IgG,AQP4-IgG), which can occasionally overlap each other, such as the combination of anti-GABABR-IgG with amphiphysin (Höftberger et al. 2013), anti-GABABR-IgG with Hu (Alexopoulos et al. 2014), anti-NMDAR-IgG with thyroid antibody (Xu et al. 2011), anti-NMDAR-IgG with AQP4-IgG (Skowrońska et al. 2019), and anti-NMDAR-IgG with MOG-IgG (Fujimori et al. 2020). Anti-NMDAR encephalitis is associated with ovarian teratoma and other tumors (Masopust et al. 2018). The incidence of tumor of anti-Amphiphysin antibody is 46 ~ 79%, which may be related to Sheehan syndrome, breast cancer, small-cell lung cancer, and angiosarcoma (Kelley et al. 2017). It is also associated with subacute sensory neuropathy, marginal encephalitis, and other paraneoplastic syndromes (Armangue et al. 2014). Anti-AMPAP antibody encephalitis is rare in clinic and was first reported in 2009, which may be associated with small cell lung cancer, breast cancer and thymic carcinoma (Höftberger et al. 2015). The detection rate of positive antibodies has increased and will continue to increase due to improved autoimmune disease detection techniques. The specific mechanism of double-positive antibodies is not clear, and may be associated with autoimmune abnormalities, gene specificity, and tumor (Tao et al. 2019). No tumor was found in our case, and the double-antibody positivity may be related to the special immune response of the body during pregnancy.

The optimal treatment of recurrent anti-NMDAR has not been determined. Pregnancy makes treatment more challenging and can lead to poor outcomes. Methylprednisolone or immunoglobulin was given in the acute phase of the first onset, and low-dose hormone or low-dose rituximab, cyclophosphamide and other second-line immunosuppressive maintenance treatments were given in the remission phase to prevent recurrence (Dalmau et al. 2008). In addition, plasma replacement may be superior to other treatments because it can be safely used in pregnancy and has antibodies that can rapidly reduce the fetal body (McCarthy et al. 2012). There is no consensus on the optimal time of termination of pregnancy and delivery mode for AE patients during pregnancy. Weighing the severity of the disease against the safety of the fetus is a major factor in determining the best time and method of delivery. A thorough assessment of the potential for cancer is crucial for anti-NMDAR encephalitis. Once found, rapid clearance may be important for induction of remission. If initial screening is negative, follow-up must be repeated. It is recommended to conduct surveillance imaging every 4–6 months for at least 4 years (Lee and Lee 2016).

## Conclusion

Pregnancy is an induction factor of anti-NMDAR encephalitis. Double positive antibodies may be associated with autoimmune abnormalities (such as pregnancy), gene specificity, and tumor. Anti-NMDAR encephalitis in pregnancy should be diagnosed in time and treated with immunotherapy as soon as possible. A timely and thorough assessment of potential cancers is essential. Once found, rapid clearance may be important for induction of remission. Those who do not find tumors should be followed up regularly for at least 4 years.
